# Extracorporeal CPR Performance Metrics in Adult In-Hospital Cardiac Arrest: A Stepwise and Evidence-Based Appraisal of the VA-ECMO Implementation Process

**DOI:** 10.3390/jcm14155330

**Published:** 2025-07-28

**Authors:** Timothy Ford, Brent Russell, Pritee Tarwade

**Affiliations:** Department of Anesthesiology and Perioperative Medicine, Medical University of South Carolina, Charleston, SC 29425, USA; fordti@musc.edu (T.F.); russebre@musc.edu (B.R.)

**Keywords:** adult, cardiac arrest, VA ECMO, in-hospital cardiac arrest, cardiopulmonary resuscitation, extracorporeal membrane oxygenation, extracorporeal cardiopulmonary resuscitation

## Abstract

Extracorporeal cardiopulmonary resuscitation (ECPR) is an established intervention for select patients experiencing refractory cardiac arrest. Among modifiable predictors of survival and neurologic recovery during ECPR implementation, timely restoration of circulation remains critical in the setting of refractory cardiac arrest (CA). The in-hospital cardiac arrest (IHCA) setting is particularly amenable to reducing the low-flow interval through structured system-based design and implementation. Despite increasing utilization of ECPR, the literature remains limited regarding operational standards, quality improvement metrics, and performance evaluation. Establishing operational standards and performance metrics is a critical first step toward systematically reducing low-flow interval duration. In support of this aim, we conducted a comprehensive literature review structured around the Extracorporeal Life Support Organization (ELSO) framework for ECPR implementation. At each step, we synthesized evidence-based best practices and identified operational factors that directly influence time-to-circulation. Our goal is to provide a stepwise evaluation of ECPR initiation to consolidate existing best practices and highlight process components with potential for further study and standardization. We further evaluated the literature surrounding key technical components of ECPR, including cannula selection, placement technique, and positioning. Ongoing research is needed to refine and standardize each stage of the ECPR workflow. Developing optimized, protocol-driven approaches to ensure rapid, high-quality deployment will be essential for improving outcomes with this lifesaving but resource-intensive therapy.

## 1. Introduction

In-hospital cardiac arrest (IHCA) remains a high-mortality event. Despite adherence to advanced cardiac life support (ACLS) protocols, survival to discharge has historically remained below 25% [1]. In recent years, extracorporeal cardiopulmonary resuscitation (ECPR) has emerged as a rescue therapy for selected patients with refractory cardiac arrest. It is increasingly recognized in international consensus statements and guidelines as a time-sensitive option for restoring circulation, particularly when delivered within an organized system with clearly defined processes and trained multidisciplinary teams [2].

Among the various predictors of outcome after cardiac arrest, low-flow time—defined as the interval between the start of conventional cardiopulmonary resuscitation (CCPR) and the establishment of adequate ECPR flow—has consistently emerged as a strong modifiable determinant of neurologic recovery and overall survival [2,3,4]. Multiple large-scale observational studies demonstrate a steep decline in favorable neurologic outcomes and overall survival as low-flow duration increases. In the context of IHCA, the best outcomes have been observed when extracorporeal membrane oxygenation (ECMO) is initiated within 20 min of CCPR. Outcomes beyond 40 min are notably poor, with diminishing likelihood of meaningful recovery despite successful ECMO deployment.

The relationship between low-flow duration and outcome is consistent with decades of resuscitation science, emphasizing the time sensitivity of cerebral perfusion. The largest contemporary analysis—a multicenter registry study of nearly 350,000 in-hospital cardiac arrest patients—demonstrated that the probability of overall survival and favorable neurologic outcome declines rapidly with increasing CPR duration. Specifically, the probability of overall survival falls below 1% after 39 min of CPR, and the probability of a favorable neurologic outcome falls below 1% after 32 min [1]. Preclinical and clinical evidence suggest that irreversible brain injury can begin to develop after as little as five minutes of no-flow or severely low-flow states [5]. In the context of CCPR, the majority of survivors with favorable neurologic outcomes achieve return of spontaneous circulation (ROSC) within 15 min, reinforcing the concept that both no-flow and low-flow time must be minimized to optimize outcomes.

Intuitively, optimizing the timing and execution of ECPR in the setting of IHCA demands a systematized approach to every step in the implementation process. Despite increasing research and broader implementation of ECPR, the literature remains limited regarding the technical and operational standards, team coordination models, and quality improvement benchmarks necessary to improve the system. While existing studies and guidelines focus on aggregate survival, they do not delineate how specific workflow decisions contribute to delays or successes across the entire implementation process. To advance the field, we must first synthesize the current evidence surrounding the ECPR implementation process in order to identify gaps, guide future research, and support the refinement of evidence-based protocols.

**Figure 1:**
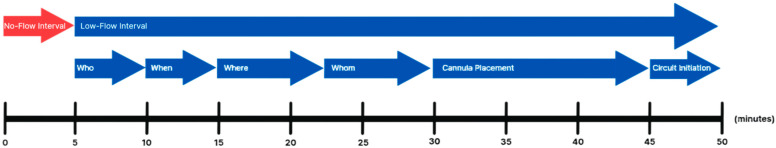
Visual Summary of Stepwise ECPR Implementation Process.

In this narrative review, we aim to evaluate and synthesize the current state of the literature on ECPR implementation for IHCA. We organize our review as a stepwise analysis of the implementation process, utilizing the framework proposed by the Extracorporeal Life Support Organization (ELSO)—namely: Who, When, Where, by Whom, Cannula Placement, and Circuit Initiation—as outlined in Figure 1. Our goal is to identify evidence-based best practices and committee consensus while highlighting components of the process that require further investigation and standardization in the context of IHCA. By mapping the ECPR workflow through this structured lens, we seek to lay the groundwork for future standardization efforts and identify actionable targets for minimizing time to circulation with ECPR in the setting of refractory IHCA.

Visual summary of the ECPR implementation process for IHCA, highlighting key sequential steps and target time intervals based on current literature. The timeline illustrates the transition from the no-flow interval to the low-flow interval, followed by critical decision points and procedural actions: team activation (“Who”), timing (“When”), location selection (“Where”), team role designation (“Whom”), cannula placement, and circuit initiation. Approximate time goals for each step are aligned proportionally to support process optimization and protocol development aimed at minimizing time-to-circulation. With respect to Where and Whom, we encompass the time of activation of the ECPR team to procedural initiation.

## 2. Methods

We performed a narrative literature review focused on the technical, procedural, and organizational components of ECPR implementation for IHCA. To identify relevant literature, we conducted a comprehensive search of PubMed, Google Scholar, and ScienceDirect. We included articles published between 1 January 2015, to present date of composition (31 May 2025). All articles were indexed in PubMed for reference generation.

Our search strategy included both broad terms and thematically focused terms to ensure a comprehensive synthesis. Broad search terms included: “ECPR,” “extracorporeal CPR,” “VA-ECMO,” “in-hospital cardiac arrest,” “cannulation,” and “ECMO implementation.” Thematic search terms specified specific implementation process steps as outlined in the ELSO framework.

The evidence was organized using a stepwise framework adapted from the ELSO Red Book, including patient selection (Who), timing (When), cannulation environment (Where, team composition (Whom), cannula placement, and circuit initiation. We included studies that discussed logistical considerations, procedural steps, or performance benchmarks in ECPR delivery.

While we primarily focused on system assessment of ECPR implementation in IHCA, we included studies on out-of-hospital cardiac arrest (OHCA) and non-arrest VA-ECMO implementation to form a comprehensive synthesis where data and guidelines on ECPR implementation in IHCA were lacking. Pediatric populations were excluded.

## 3. Who

The use of ECPR in refractory cardiac arrest is supported by growing evidence [6,7]. However, initiating ECPR remains a complex, resource-intensive intervention that must be reserved for carefully selected patients [1,8]. Candidate selection is a vital step in the implementation process, as choosing inappropriate candidates may expose patients to invasive support with a limited chance of recovery. Despite increasing global adoption, there remains considerable variability in eligibility criteria for ECPR candidacy across studies. Lenient inclusion criteria may increase access but are associated with lower rates of favorable outcomes, while overly restrictive protocols risk excluding patients who may have benefited [9]. Importantly, ECPR candidacy determination is a time-sensitive process performed in a chaotic environment where knowledge about the patient may be minimal; thus, understanding the most important factors in candidate selection theoretically will help streamline this first step in the process.

Contraindications for ECPR are generally present when ECPR will not successfully bridge to recovery or alternative destination therapy, such as a durable mechanical circulatory assist device, or when meaningful neurologic recovery is not expected despite circulatory restoration. Candidates may also be deemed ineligible for practical reasons, such as technical limitations (e.g., high BMI) or if ECMO is not aligned with the patient’s wishes [10].

Multiple factors considered in candidate selection can be enumerated as follows:

### 3.1. Age

Age limit remains one of the most variable factors in research protocols. Reported cutoffs range from 50 to 80 years, with 75 years being the most common among protocols. There is a general consensus that higher age is associated with poorer outcomes. In 2024, the AHA criteria recommended initiation of ECPR in patients aged less than 75 years [11].

### 3.2. Witnessed vs. Unwitnessed Cardiac Arrest

Witnessed arrest is another common criterion used to determine ECPR candidacy. According to a review by Alenazi et al., approximately 68% of systems included witnessed cardiac arrest as part of their selection criteria [11]. Unwitnessed cardiac arrests often involve unknown or prolonged no-flow intervals, which are associated with poorer neurologic outcomes and increased mortality.

### 3.3. Initial Rhythm

Shockable rhythms—ventricular tachycardia (VT) and ventricular fibrillation (VF)—are considered strong predictors of improved outcomes [12]. They are typically associated with shorter no-flow intervals compared to non-shockable rhythms such as pulseless electrical activity (PEA) or asystole. According to Alenazi et al., 30% of systems included initial rhythm as part of their selection criteria for determining ECPR candidacy. Pozzi et al. modified their institutional protocol to only include shockable rhythms for ECPR, resulting in an increase in the rate of favorable neurologic outcome from 4.4% to 23.5% [13].

### 3.4. Presence of Comorbidities

The goal of ECPR is to bridge patients in cardiac arrest to recovery, heart transplant, or to durable mechanical circulatory devices such as the left ventricular assist device (LVAD). Patients with chronic end-stage organ dysfunction or malignancy are usually not considered candidates for ECPR. Bourcier et al. eliminated patients older than 80 years, with preexisting multiorgan dysfunction, ventilator dependency (defined as greater than one month), prolonged immobility (defined as bedridden for greater than one month) or loss of independence, intracranial hemorrhage or infarct, uncontrollable bleeding, or terminal stage malignancy [14]. Serum lactate levels greater than 5 mmol/L have been associated with poor candidate prognosis. An analysis of the ELSO registry showed a 2.3-fold increase in mortality after ECPR in patients with a BMI greater than 40 kg/m^2^. Dilated pupils are also a sign of poor prognosis, and cerebral near-infrared spectroscopy (NIRS) is another potential way to assess prognosis for ECPR [15]. While brain NIRS is usually not available at bedside on the general wards, it can be used in or upon transfer to the intensive care unit (ICU) and should be considered when available.

### 3.5. Potential Organ Donation

In addition to the aforementioned indications, “potential organ donation” has also been considered a justification for proceeding with ECPR. In a recent retrospective study that study of 307 OHCA patients, in-hospital mortality was 83% (256 patients). Among these, solid organ donation occurred in 19% (58 patients). According to this study, as criteria for ECPR implementation became increasingly restrictive, the solid organ donation rate declined from 19% in the overall cohort, to 16% when low-flow time was less than 60 min, and further to 11% when low-flow time was less than 60 min and the initial rhythm was shockable rhythm [16]. This is in alignment that patient outcomes were better with lower low flow time and shockable rhythm. With restrictive candidate selection, increased survival leads to fewer patients and organ donation.

In Prague, a randomized controlled trial was performed on refractory OHCA of cardiac origin. Patients were randomized to ECPR or standard management with the aim of studying the effect on organ donation rate and one year organ survival. The ECPR-based approach demonstrated an increased rate of organ donation and excellent outcomes of transplanted organs [17]. Although these findings were derived from OHCA, we believe these results may also have relevance to the in-hospital cardiac arrest setting and can be considered as part of broader discussions around ECPR candidacy.

## 4. When

No flow time—Time from cardiac arrest to start of chest compression is considered as no flow time. No flow time cutoffs vary from zero to 20 min, and less than 5 min is the most frequently utilized [11].

Low flow time—Low flow time can be defined as the time from initiation of chest compressions to the start of ECMO flow. In recent consensus, ECPR is recommended for patients in whom ROSC is not achieved within 10–15 min and had no contraindications to ECPR [18]. Low flow time cut off varies from 15 min to 150 min–60 min being the most common cutoff [11]. It is important to consider that to initiate the ECMO flow within that time, ECPR candidacy should be discussed within 10–15 min of ECPR implementation [19]. Per Wengenmayer et al. survival rates were 67% in patients (*n* = 14) with a CPR duration shorter than 20 min and 29% (*n* = 33), 10% (*n* = 43), and 6% (*n* = 43) after 20–45, 45–60, and 60–135 min of mechanical CPR, respectively. Sim et al. performed a retrospective study which compared low flow time <20 min, 20–40 min and >40 min and neurological outcomes were significantly better with lower low flow time at 3 months and 6 months [4].

## 5. Where

Patients can be cannulated at the bedside or in an interventional catheterization lab. For an outside hospital arrest, “scoop and ride” or “stay and play” approaches are suggested [20]. According to a multicenter prospective study in North America—a “stay and play” strategy has no added benefit beyond CPR time beyond 15–20 min [21]. Continuing good quality CPR during transport reduces the risk caused by interruption of CPR. A cohort study of 2515 patients from the ELSO registry showed overall mortality was 67.6%, with higher mortality found in patients in ICU and acute care hospital beds. In addition, it found that moving the patient from their initial location to a secondary location, such as a catheterization lab for ECPR cannulation, did not give any additional benefits [22].

## 6. Whom

ECPR demands the rapid orchestration of a highly trained multidisciplinary team. Most programs include a cardiac surgeon or interventional cardiologist, intensivist or anesthesiologist, perfusionist, ECMO-trained registered nurse, and increasingly a clinical pharmacist. Several institutions report improved outcomes with the inclusion of a full-time intensivist and ECMO pharmacists within the team structure, ensuring around-the-clock expertise and coordinated decision-making [23].

Team structure and responsibilities ultimately depend on institutional protocols and system design. Coordination requires a highly trained team to run parallel tasks of managing ongoing cardiopulmonary resuscitation while simultaneously coordinating ECPR implementation logistics.

## 7. Cannula Placement

### 7.1. Cannula Site Selection

Current guidelines from major societies emphasize the femoral vessels as the recommended site for VA-ECMO cannulation in the setting of ECPR. This preference is based on the importance of rapid access, the feasibility of percutaneous cannulation, and the ability to initiate support outside of procedural suites [24,25]. This approach is particularly advantageous in emergent settings where minimizing the low-flow interval is critical. Bilateral cannulation is preferred due to the associated lower risk of compartment syndrome, bleeding, and in-hospital mortality [26].

Alternative cannulation sites include the axillary and subclavian arteries. Upper arterial access offers theoretical advantages, including reduced risk of differential hypoxemia and facilitating patient mobilization. However, these approaches are associated with increased procedural complexity, higher bleeding risk, potential hyper-perfusion of the ipsilateral upper extremity, and a greater incidence of vascular complications [27,28]. Ultimately, cannulation site selection should be individualized, accounting for patient anatomy (e.g., morbid obesity, severe iliofemoral disease, or prior vascular surgery), clinical context, and the expertise of the cannulating team [25,29,30]. Importantly, there are no randomized controlled trials directly comparing cannulation site strategies, with most recommendations based on observational data and expert consensus.

From a quality improvement perspective, performance metrics related to site selection are inherently limited, given the patient-specific nature of these decisions. However, we propose tracking the frequency and distribution of cannulation sites across ECPR cases as a useful system-level metric. Monitoring this distribution may help identify trends, operator preferences, deviations from protocol, and facilitate retrospective analysis to inform future research and guideline development regarding optimal cannulation site selection.

### 7.2. Cannula Size Selection

Effective ECPR relies heavily on appropriate cannula selection to optimize circuit flow and minimize complications. Current adult practice typically involves the use of venous drainage cannula ranging from 21 to 29 French (Fr) and arterial return cannula between 15 and 19 Fr [24]. The decision regarding cannula size should be individualized based on patient-specific factors, including vessel caliber, body surface area (BSA), and relatedly the anticipated flow requirements necessary to maintain adequate end-organ perfusion.

A recurring pattern observed in the literature is that smaller cannulas are more frequently employed in bedside ECPR settings, while larger cannulas are more commonly used in environments such as the catheterization laboratory or operating room [24,31]. This variation likely reflects the increased comfort of proceduralists with image-guided techniques, which facilitate the placement of larger cannulas with greater precision. Despite the higher flow potential offered by larger cannulas, existing evidence suggests that comparable clinical outcomes may be achieved using smaller cannulas, with the added benefit of a reduced complication profile. This includes lower rates of distal limb ischemia, bleeding, vascular trauma, and infection [24]. Concerns of increased risk of hemolysis as a complication of smaller catheter sizes are not supported by the literature [32,33]. Although further research is warranted to determine the optimal cannula size relative to physiologic and anatomic variables, current data support a strategy of selecting the smallest cannulas capable of achieving sufficient flows, thereby balancing the goals of perfusion adequacy and complication minimization.

From a systems-based standpoint, incorporating performance metrics related to cannula size into ECPR quality improvement efforts may enhance program readiness and response efficiency. While time-based performance metrics specific to cannula sizing are limited, readiness metrics can be readily implemented. We propose routine daily audits to verify the availability of a full range of sterile arterial and venous cannula sizes, ensuring preparedness for the unpredictable and time-sensitive nature of ECPR deployment. Furthermore, although cannula sizing is often nuanced and subject to provider judgment, we propose the adoption of standardized protocols that encourage the use of the smallest cannula size capable of delivering target flows. Specifically, cannula selection should aim to support a cardiac index of 2.2 to 2.4 L/min/m^2^, guided by calculated or estimated BSA [24]. While precise determination of vessel caliber and anatomic suitability may be limited in emergent situations, this approach may promote consistency in clinical practice while supporting both timely cannulation and the reduction in complications. Additional avenues for performance tracking may include retrospective audits correlating cannula size to achieved flow rates based on BSA and complication incidents. Over time, such data could refine institutional best practices and help benchmarking across ECPR centers.

### 7.3. Cannula Insertion

While surgical cutdown is associated with lower rates of vascular complications, percutaneous cannulation is generally favored in the ECPR setting due to its association with shorter flow initiation [34,35]. Given that the low-flow interval Is among the few modifiable predictors of survival and neurologic outcome, prioritizing techniques that expedite cannulation is critical to optimizing patient outcomes [36].

Percutaneous access can be performed using either surface anatomical landmarks or image guidance. Landmark-based techniques have been associated with increased rates of cannula malposition and prolonged cannulation times when compared to image-guided approaches. In contrast, ultrasound guidance has consistently demonstrated improved first-pass success rates, reduced mechanical complications, and shorter time to cannulation [30,37,38,39]. Fluoroscopy offers the advantage of real-time confirmation of guidewire and cannula position, particularly in patients with complex or atypical anatomy. However, it has not shown a consistent time advantage over ultrasound guidance [40]. Moreover, the need to transport a patient undergoing active conventional CPR to a fluoroscopy capable procedure suite can introduce delays and compromise the quality of chest compressions during transit, negating the theoretical benefit in procedural accuracy [41].

In the context of ECPR program development and quality improvement, we propose that cannulation techniques should be assessed through a dual lens of readiness and time-based performance. Readiness metrics should include daily verification of functional ultrasound machines, consistent availability of sterile probe covers and vascular access kits, and established protocols to ensure that all necessary equipment is immediately accessible wherever cannulation is anticipated. Time performance metrics may include the measurement of skin-to-wire and skin-to-cannula intervals, which provide granular insights into procedural efficiency. Monitoring these metrics over time enables identification of workflow bottlenecks and guides targeted interventions such as simulation-based training, staffing model optimization, and equipment standardization.

### 7.4. Cannula Position Confirmation

Accurate and timely placement of the venous drainage and arterial return cannulas is essential for ensuring effective flow and minimizing complications [24,42,43]. In the context of peripheral femoro-femoral ECPR, the venous cannula should ideally be advanced into the inferior vena cava at the junction of the right atrium to optimize preload reduction in circuit drainage. The arterial cannula tip should be positioned within the iliac or distal abdominal aorta and oriented cephalad to ensure effective retrograde aortic perfusion.

Two primary imaging modalities are employed to confirm proper cannula positioning: transesophageal echocardiography (TEE) and fluoroscopy. Fluoroscopy is commonly utilized in catheterization laboratories to guide both guidewire and cannula advancement, offering real-time confirmation of vascular trajectory and positioning. By contrast, in emergency or ICU based ECPR scenarios where fluoroscopy is often unavailable and rapid cannulation is essential, TEE provides a practical and effective alternative. Indeed, TEE offers high-quality, real-time visualization of cannula placement without interrupting ongoing chest compressions or delaying circuit initiation [44,45,46,47].

The literature supports the use of both TEE and fluoroscopy, with TEE offering utility for visualizing venous cannula position, confirming right heart decompression, and excluding complications such as intracardiac thrombus or cannula mal-position. With respect to arterial cannula confirmation, while TEE is limited with respect to direct visualization of the peripheral arterial return cannula tip, it can be utilized to both confirm guidewire passage into the descending aorta and exclude wire malposition in the cardiac chambers [24,47,48]. Although no randomized controlled trials definitively favor one modality over the other, expert consensus emphasizes the importance of employing some form of real-time imaging to confirm cannula location as a standard practice. Transesophageal echocardiography has been highlighted in multiple feasibility studies and consensus statements for its ability to be rapidly deployed, maintain image quality during active compressions, and minimize hands-off time [49]. Accordingly, major guidelines recommend TEE as the preferred modality for cannula confirmation when available with transthoracic echocardiography (TTE) or fluoroscopy considered acceptable alternatives. At minimum, bedside ultrasound guidance is considered a foundational standard for placement.

From a quality improvement perspective, we propose tracking imaging utilization rate defined as the proportion of ECPR cases in which TEE, TTE, or fluoroscopy is employed to confirm cannula positioning intuitively serves as a valuable performance metric. Routine tracking of this measure can help ensure imaging protocols are consistently followed, and that training gaps or equipment deficiencies are rapidly identified. Furthermore, we propose skin-to-cannula-position confirmation time as a time performant metric to monitor team responsiveness and procedural efficiency across varied clinical environments.

Adding opportunities for system optimization may include implementing standardized imaging protocols within ECPR checklists, conducting regular multidisciplinary simulations focused on cannula placement and confirmation, or integrating post case debriefs that explicitly review imaging timelines and findings. These efforts may help promote consistency across operators to ensure imaging is both timely and accurate in addition to supporting institutional learning.

### 7.5. Cannula Placement Associated Complications

Complications related to ECMO cannulation during ECPR represent a significant source of morbidity and mortality. While these complications may arise from each distinct phase, namely cannula size selection, insertion, and positioning, they frequently overlap. The Extracorporeal Life Support Organization provides a standardized framework for classifying ECMO-related complications as either medical or mechanical, which has been widely adopted in both clinical practice and research reporting.

Medical complications related to ECMO cannula placement are the most prevalent and include infection, bleeding, vascular injury (e.g., dissection, perforation, pseudoaneurysm), limb ischemia, compartment syndrome, and rarely cardiac tamponade. Among these complications, bleeding and limb ischemia are especially common in the context of ECPR. Bleeding rates associated with cannulation may range from 15 to 46%, while limb ischemia occurs up to 20%, and infection rates as high as 11%, and cardiac tamponade at ~2–3% [40,42,43,44,45,46,47,50]. Access site selection also impacts complication risk, with higher bleeding rates reported in axillary and subclavian approaches compared to femoral access [17,18].

Mechanical complications related to cannula placement include cannula dislodgement, kinking, inadequate sizing, migration, and malposition. Improper cannula positioning can result in inadequate venous drainage, insufficient arterial flow, or increased circuit pressures, all of which compromise support.

Many of these complications are reducible through adherence to evidence-based practices. Indeed, expert consensus emphasizes standardization and protocolization of all phases of cannula placement. This includes bilateral femoro-femoral site selection, utilizing the smallest cannula capable of achieving adequate flow, employing image-guided percutaneous cannulation, and confirming position with transesophageal echocardiography or fluoroscopy. Consistent application of these strategies, supported by structured team training and performance review, can reduce procedural risk.

To ensure ongoing improvement, routine surveillance of complications is essential. Participation in structured quality reporting platforms, such as the Get With The Guidelines-Resuscitation (GWTG-R) for IHCA and the ELSO Quality Reporting Platform for ECMO-related complications, enables institutions to benchmark outcomes, receive feedback, and guide targeted quality improvement efforts [51,52]. The ELSO registry, the largest international ECMO database, provides a standardized framework for tracking ECPR-related complications. Transparent reporting not only supports institutional learning but also contributes to broader efforts aimed at improving ECPR safety and efficacy across centers.

## 8. Circuit Initiation

Initiating extracorporeal circulation during ECPR is a time-sensitive and precise process that marks the transition from vascular access to active hemodynamic support. Once appropriate cannula placement is confirmed, the next step involves connecting the patient to the ECMO circuit to reestablish systemic circulation. This process comprises 3 essential steps: verifying correct inflow and outflow line orientation to avoid reversal, performing a sterile wet-to-wet connection to maintain circuit integrity and minimize air entrainment, and initiating and confirming adequate circuit flow to restore circulation [7,23].

Currently, there are few metrics to help guide the measurement and continuous improvement of this process. However, if each of these steps is unstandardized or delayed, they can theoretically introduce avoidable inefficiencies that prolong the low-flow interval [53]. As such, optimization of circuit initiation represents an important systems level target for both training and continuous performance monitoring.

In alignment with prior sections, we once again propose a dual categorization of tracking metrics for circuit initiation. With respect to system readiness metrics, we propose routine daily audits to verify a pre-primed ECMO circuit is dedicated to ECPR, development of and adherence to a standardized protocol for confirming inflow and outflow line identification and connection, and development and adherence to a wet-to-wet connection protocol. Each of these elements should be subject to structured documentation and ongoing audit to ensure that ECPR teams remain equipped and aligned in the event of ECPR system activation.

Time-performance metrics may include the interval from confirmed cannula placement to wet-to-wet circuit connection, and from cannula position to achieve full flow verification. This data can be tracked alongside clinical outcomes to identify bottlenecks, assess team performance, and guide simulation training or protocol revisions. The incorporation of standardized checklists and cognitive aids to promote reliability during circuit initiation.

Complications related to circuit initiation include but are not limited to air embolism, connection errors, raceway or circuit rupture, heat exchanger malfunction, oxygenator failure, and pump malfunction [53]. Consistent reporting of these events to the ELSO Quality Reporting Platform supports institutional learning and contributes to global benchmarking efforts aimed at improving safety and reliability in ECPR.

## 9. Conclusions

ECPR is a complex but potentially life-saving intervention for select patients experiencing refractory IHCA, with low-flow duration emerging as the most consistently impactful and modifiable predictor of outcome. Although many protocols cite 60 min as an upper threshold for acceptable low-flow duration, recent data suggest that this target may be insufficient to see significant outcome improvement, particularly at experienced centers. Enhancing procedural efficiency requires structured system readiness supported by strategies such as workflow simulation, system audits, and performance tracking. Table 1 outlines key system-level metrics that can guide quality improvement across ECPR process implementation. Multicenter standardization of these operational metrics could support collaborative learning, ensure consistent practice, and ultimately improve patient outcomes. The future of ECPR lies in refining the systems that deliver it, with a persistent emphasis on time, team, and technical readiness.

Summary of proposed performance metrics for ECPR implementation in the setting of in-hospital cardiac arrest (IHCA), organized by process step. Quality metrics reflect patient-level characteristics that influence candidacy and outcomes, including age, rhythm, comorbidities, and estimated low-flow interval. Readiness metrics evaluate system preparedness, such as point-of-care availability, team designation, and equipment readiness. Time-performant metrics quantify key procedural intervals, including cannula placement and circuit initiation benchmarks. Together, these metrics provide a structured framework for continuous quality improvement and standardized performance evaluation in ECPR programs.

## Figures and Tables

**Table 1:** Summary of proposed performance metrics.

Process Step	Quality Metrics	
Who	Age	<75 years
	Cardiac Arrest	Witnessed
	Rhythm	Shockable
	Comorbidities	Absence of end stage or terminal disease
	Lactate	<5
	BMI	<40 kg/m^2^
	Estimated no-flow interval	<5 min
	Low-flow interval	<60 min
When	Discuss candidacy	10 min
Where	Point of Care	Yes
Whom	Designated Institutional Team	Yes
	**Readiness Metrics**	**Time Performance Metrics**
Cannula Placement		
Site Selection	Site Assessment by Ultrasound	
Size Selection	Cannulas Readily Available	
Insertion	Insertion by Ultrasound Guidance	Skin-to-wire time, Skin-to-cannula time
Position Confirmation	TEE or Fluoroscopy Available	Time to Position Confirmation
Circuit Initiation	ECMO Pre-Primed Circuit	Wet-to-Wet Connection Time
	Wet-to-wet Protocol	Time to Targeted Flow

## Data Availability

No new data were created or analyzed in this study. Data sharing is not applicable to this article.

## Abbreviations

The following abbreviations are used in this manuscript:

ECPR	Extracorporeal Cardiopulmonary Resuscitation
CA	Cardiac Arrest
IHCA	In-Hospital Cardiac Arrest
ELSO	Extracorporeal Life Support Organization
ACLS	Advanced Cardiac Life Support
CCPR	Conventional Cardiopulmonary Resuscitation
ROSC	Return of Spontaneous Circulation
ECMO	Extracorporeal Membrane Oxygenation
OHCA	Out-of-Hospital Cardiac Arrest
VT	Ventricular Tachycardia
VF	Ventricular Fibrillation
LVAD	Left Ventricular Assist Device
